# The Community Cost of Maintaining Gastrostomies in Pediatric Patients

**DOI:** 10.1097/PG9.0000000000000278

**Published:** 2022-12-30

**Authors:** Jasmine Makker, Caroline Pardy, Veronica Kelly, Iain Yardley

**Affiliations:** From the * GKT School of Medical Education, King’s College London, London, United Kingdom; † Department of Paediatric Surgery, Evelina London Children’s Hospital, London, United Kingdom; ‡ Department of Paediatric Neurosciences, Evelina London Children’s Hospital, London, United Kingdom; § The Mary Sheridan Centre, Evelina London Children’s Community Services, London, United Kingdom.

**Keywords:** public health, financial, economic

## Abstract

The use of gastrostomies in children is increasing and their need for long-term management following insertion presents a potentially significant financial and resource challenge to local healthcare systems.

**Objectives:**

The aim of this study was to determine the yearly cost of maintaining a gastrostomy in a pediatric patient.

**Methods:**

A retrospective, bottom-up cost-analysis was conducted in a cohort of 180 patients with gastrostomies aged 0–19 years. One in 5 patients were randomly selected for individual cost analysis (n = 36). Their electronic health record was interrogated from the period of March 1, 2019–March 1, 2020. Costs included in the analysis were staff contact time from the community nursing and nutrition teams, and equipment costs.

**Results:**

The mean cost of maintaining a pediatric gastrostomy across all ages was £709.87 (SD 403.18) per year. Mean annual cost varied by age, underlying diagnosis, and gastrostomy device, but this variation was only statistically significant for the type of device, with Mic-Key buttons having a mean annual cost of £834.66 (SD 307.85), Mini buttons £799.06 (SD 395.01), and percutaneous endoscopic gastrostomy tubes £279.34 (SD 297.45; *P* = 0.004).

**Conclusion:**

The mean cost of maintaining a gastrostomy in a pediatric patient is just over £700 per year. The cost is the highest as a child enters adulthood. Button devices have higher maintenance costs compared with percutaneous endoscopic gastrostomy tubes.

**What Is Known**

- The prevalence of gastrostomy in a general pediatric population is 84 per 100 000 children.
- The increase in the use of gastrostomies and the required ongoing management represents a significant resource and financial challenge to local healthcare systems.
- The absolute costs associated with the care of a gastrostomy are not well defined with wide variation in estimates in published studies.

**What Is New**

- The community cost of maintaining a gastrostomy in a child is just over £700/year.
- The cost is the highest just as patients enter adulthood.
- Costs vary according to the device used; standardizing care may have the potential for cost savings.

## INTRODUCTION

Enteral feeding is used to support nutrition in children, most commonly due to neurological disorders, followed by malnutrition, and structural abnormalities.^1^ A gastrostomy facilitates feeding in these children, has been shown to improve nutrition and growth, and is associated with high caregiver satisfaction.^2–4^

Various studies have detailed the insertion costs of a gastrostomy.^5,6^ The national average costs for the insertion of a gastrostomy and the subsequent inpatient stay are £1940 for patients under 18 years of age, and £917 for those aged 19 and over.^7^ The management of complications following insertion also generates considerable cost. The rate of complications associated with a gastrostomy varies widely in the literature, ranging from 2.0% to 14% for major complications (requiring hospitalization or additional interventional procedures), and 11.4%–56% for minor complications such as granulation, local infection, and leakage.^8–11^ The resulting contact with the healthcare team represents an area of significant resource utilization. Two studies conducted in the United States showed average costs of 1200^12^ and 913.10 USD,^13^ respectively, for each visit to the emergency department pertaining to gastrostomy-related complications.

Previous studies of cost-analysis for gastrostomy have focused on a specific age range of the pediatric population,^6,14^ or a particular indication for gastrostomy insertion.^15^ Currently, data detailing the cost of maintaining a gastrostomy in the general pediatric population are not known. This information would be useful to both policymakers and healthcare providers, enabling identification of the highest areas of the cost associated with gastrostomy, as well as establishing potential areas for cost reduction. The aim of this study was to determine the cost of maintaining a gastrostomy in pediatric patients for a period of 1 year.

## METHODS

### Study Design and Study Population

The study was approved by the lead author’s institution as a service evaluation (Reference number 11677). A pediatric surgeon, clinical nurse specialist, home enteral nutrition (HEN) dietician, and community nurse were interviewed to establish the patient pathway following gastrostomy insertion at a tertiary pediatric center in London.

A retrospective cost analysis of maintaining a gastrostomy was conducted on a cohort of 180 children aged 0–19 years in 3 London boroughs (Southwark, Lewisham, and Lambeth), who had a gastrostomy in situ during the period from March 1, 2019–March 1, 2020. The local point prevalence of gastrostomy in these boroughs during this period has previously been established.^1^ To perform a cost analysis, a 20% sample was randomly selected from each of the following age groups (0–4, 5–9, 10–14, and 15–19 years). Patients were excluded and replaced with an alternative randomly chosen patient if there was a lack of data on the community medical record database or if the patient was transferred to the care of a Clinical Commissioning Group (CCG) in a different borough during the study period.

### Data Collection

Data detailing patient demographics (age, sex, date of birth) as well as the primary diagnosis, type of gastrostomy device originally inserted, date of insertion, and indication for insertion were obtained from the patient’s electronic records. Data for direct and indirect patient contact by the community medical team were collected from the community pediatric healthcare database. These visits were then categorized as: surveillance, replacement of a device, consultation due to complications, height and weight assessments, training of school staff and family members, and prescription and supplies. Data relating to changes in feeding care plans and HEN dietician visits were acquired from the HEN team database. For these interactions, if exact times were not known, a telephone call was assumed to take 15 minutes and a home visit 45 minutes.

### Cost Evaluation

The costs identified were classified as equipment or labor costs. Insertion costs were not accounted for, as the focus of this study was the cost of maintaining a gastrostomy. Unit costs for all required materials and equipment were derived from local rates agreed by the CCG of Southwark, Lewisham, and Lambeth. Staff costs were calculated by determining the grades of staff involved in each interaction, relating this grade to the hourly rates described in the 2019/20 NHS Terms and Conditions of Service Handbook, adding high-cost area supplements for inner London, and multiplying by the time spent.^16^

### Data Analyses

Summary statistics were used to describe patient characteristics and costs of care. Patients were stratified by age group, gastrostomy device in situ, and the primary diagnosis. Univariate analysis was performed with ANOVA to identify differences in cost within the groups.

## RESULTS

### Patient Demographics

One-hundred and eighty patients were identified to have a gastrostomy in situ in the boroughs of Southwark, Lewisham, and Lambeth. Thirty-six patients were randomly selected from within their age groups. Eight of these were excluded due to lack of follow-up data or transfer of care to a different CCG, and were replaced by another randomly selected patient. Complete data for 36 patients were analyzed.

### Follow-Up Visits

**FIGURE 1.**
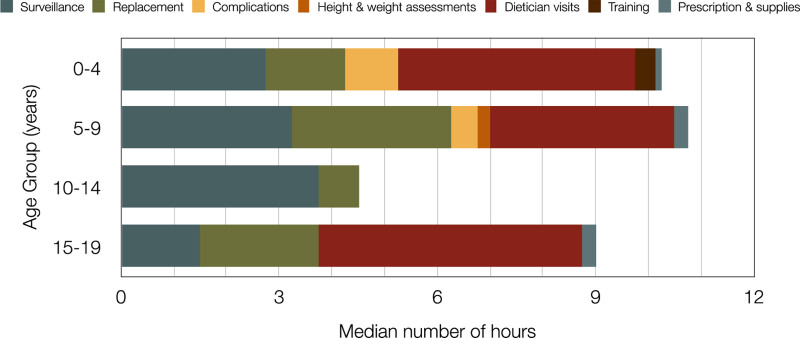
The median time spent on the different types of visits for the 4 age groups.

The median total contact hours with the patient for the different age groups over a period of one year were: 10.25 hours for 0–4 years, 10.75 hours for 5–9 years, 4.5 hours for 10–14 years, and 9 hours for 15–19 years. The median contact time distributed by type of visit for the 4 different age groups is summarized in Figure 1. Visits relating to surveillance, replacement, complications, and prescriptions were made by the Community Nursing Team. Height and weight assessments, and training were performed by Complex Needs Specialist school nurses. Visits relating to feeding care plans were made by the HEN dieticians. All community nurses and dieticians included in community care were Band 6 or 7 of the NHS Pay Scale with hourly costs ranging from £17.80 to £25.86, depending on the qualifications of the healthcare worker and their years of experience.

### Costs Identified

The mean annual cost of maintenance per gastrostomy was £709.87 (SD 403.18). Total costs stratified by the different age groups, type of gastrostomy device, and underlying condition which prompted the gastrostomy insertion are summarized in Table 1. On univariate analysis, differences in overall cost only reached statistical significance for the type of gastrostomy device (*P* = 0.004), with percutaneous endoscopic gastrostomy (PEG) devices being significantly cheaper than button devices. This variation was entirely due to differences in the equipment costs. The cost burden on the community was £588 and £586/year for Mini (Applied Medical Technology, Inc., Brecksville, USA) and Mic-Key buttons (Halyard Health Inc, Atlanta, USA), respectively, compared with just £7.41/year for PEG devices.

**TABLE 1.** Costs of maintaining a gastrostomy per patient per year, by age, device, and diagnosis

	Mean total costs/year (SD) (£)	Mean staff costs/year (SD) (£)
Age group, y		
0–4	525.44 (330.33)	246.80 (127.37)
5–9	867.05 (341.71)	249.37 (124.46)
10–14	551.55 (388.33)	146.75 (158.25)
15–19	918.18 (533.64)	244.50 (129.17)
Gastrostomy device		
Mic-Key button	834.66 (307.85)	228.76 (107.66)
Mini button	799.06 (395.01)	225.68 (156.49)
PEG tube	279.34 (297.45)	203.49 (146.63)
Primary diagnosis		
Neurological	794.51 (587.08)	224.54 (189.77)
Malnutrition	754.09 (273.65)	257.72 (92.04)
Structural	710.19 (152.97)	216.94 (46.60)
Other	647.89 (433.82)	202.95 (151.38)

PEG = percutaneous endoscopic gastrostomy.

## DISCUSSION

We report the first study of the costs of maintaining a gastrostomy in a general UK pediatric population at the individual patient level. We find the average cost of maintaining a gastrostomy in the community to be just over £700/patient/year. Community healthcare team staff costs account for just over a third of these costs and the remainder arise from material costs. Costs vary significantly according to the type of gastrostomy device in situ, with PEG tubes being much cheaper to maintain than button devices due to greatly reduced material costs.

Differences in mean costs between the different age groups were largely attributable to the type of gastrostomy. The lowest cost was in the youngest children (0–4 years), in whom 5 of 9 children had a PEG in situ. The highest cost was identified in the 15–19 years age group. All 5 patients in this group had a button gastrostomy in situ. However, due to the small number of children representing this age group, our results may not be reflective of a larger population. One child in this age group with an underlying neurological diagnosis required many more button changes, resulting in an annual equipment cost of almost 3 times that of the other children in this age group. If this child was excluded from our results, the mean annual cost of maintaining a gastrostomy would be highest in the 5–9-year age group, as might be anticipated.

Cost was highest in children with an underlying neurological disorder, but this was not significant, and there was little difference in cost related to primary diagnoses (Table 1). The increased cost in children with an underlying neurological diagnosis was attributable to equipment cost, with no increased staff cost identified in this subgroup.

Staff costs related to complications were highest in the 0–4-year age group, followed by the 5–9-year age group. This is likely to be a result of more challenging gastrostomy replacement while the new gastrostomy tract is maturing in the youngest patients, reduced compliance of the younger children related to age, and reduced experience of the carer in managing a newer gastrostomy. Staff costs related to dietetic input was fairly evenly spread over the age groups (Fig. 1), with the exception of the 10–14-year age group, which appears to represent a period of stability related to nutrition. This was also the age group with the lowest total staff costs, reflecting the reduced dietetic input required at this age, as well as reduced complications.

The overall annual cost of maintenance in our cohort is somewhat lower than, but comparable to, that reported by a 2002 UK study of children with neurodevelopmental disabilities, this estimated a weekly price of £19, or just under £1000 per year.^15^ The difference is smaller when only children with neurological disorders in our study are considered, their costs were slightly higher than other diagnoses at £831/year in our study. The remaining difference in cost could be accounted for by the increasing use of gastrostomies over time,^17^ decreasing cost of equipment, or variation in negotiated equipment unit cost between different CCGs. Two studies from the United States detailing the cost of maintenance of a gastrostomy found significantly higher costs than our study with both reporting costs over US$40 000/year.^19,^^20^ It is, however, unlikely that these costs are reflective of those in the United Kingdom, due to fundamental differences in the structure of healthcare systems making international comparisons difficult. A further UK-based study^18^ demonstrated that a Nutritional Support Team (the equivalent of HEN) reduced costs to the healthcare service by 21% for each patient with a gastrostomy in an adult population. They reported a median contact time with the Nutritional Support Team of 130 minutes, lower than the 270 minutes median contact time with the HEN in our cohort. The increased time required in our pediatric population is likely to be related to the more complex nutritional requirements of children as they grow and develop and the increased requirement for more detailed communication with a patient and their carer compared to an adult patient.

One of the strengths of our study is its bottom-up approach for cost analysis. This facilitates the evaluation of patient-specific direct costs and is a reliable method of ascertaining costs at the individual patient level.^19^ However, we limited our cost estimates to direct costs, including labor and equipment costs. Indirect costs, such as productivity losses for the parents, were beyond the scope of this study. According to Heyman et al,^20^ the cost of home care by the primary caregiver, including economic and psychological costs, for a child with a gastrostomy tube was US$37 232 a year. In addition, the cost of feeding was also not taken into account as it was considered to be similar to that of a normal oral diet, but a 2015 UK study calculated the daily costs of enteral feeding at £7.41 per patient,^21^ more than twice the national daily expenditure on food at that time.^22^ For these reasons, our cost evaluation is likely to be an underestimation of the actual cost. It should also be noted that we only considered community costs, in-hospital costs will also be a significant portion of the overall cost of a gastrostomy, and savings in the community may impact acute care costs and vice-versa. This is particularly relevant to the type of device in situ, a PEG tube requires changing in a hospital setting, albeit much less frequently than a balloon device, which transfers the costs from community to acute settings and likely accounts for much of the lower community costs seen with PEG tubes. Additionally, our study was carried out in 3 inner London boroughs. This has obvious implications for labor costs due to the addition of a London supplement in the national terms and conditions for NHS staff, but also potentially has less obvious implications due to the underlying demographics and socioeconomic status of the population, transport links, supply chains, and so on. Extrapolation to a wider pediatric population should be made with caution.

Further study is needed to provide a more accurate insight into the ongoing community cost of maintaining a gastrostomy nationally. This could usefully entail a much larger, prospective, multicenter observational study. Differences in cost should be examined in detail to explore where potential cost savings and efficiencies lie.
